# Truncal Acne: Pathophysiology, Clinical Features, and Management Strategies

**DOI:** 10.1111/jocd.70715

**Published:** 2026-02-13

**Authors:** Xiaoyue Feng, Yong Chen, Youting Liu

**Affiliations:** ^1^ R&D Department, Beijing Uproven Medical Technology Co., Ltd. Beijing China; ^2^ Beijing Uproven Institute of Dermatology Beijing China

## Abstract

**Background:**

Truncal acne is common but underrecognized. Its pathogenesis is often considered similar to facial acne, so most studies and guidelines focus on facial involvement despite the substantial disease burden of truncal lesions.

**Objective:**

This narrative review compares structural and physiological differences between truncal and facial skin, evaluates their impact on acne pathogenesis, and analyzes advantages and limitations of topical and systemic therapies, highlighting the role of routine skincare.

**Methods:**

PubMed and Google Scholar were searched for English‐language articles published up to December 2025 using keywords with Boolean operators. Evidence was narratively synthesized, and major study limitations were summarized.

**Results:**

Truncal skin has a thicker stratum corneum, lower sebaceous gland density and activity, and site‐specific differences in pH, sweat gland distribution, and microbiota, influenced by clothing occlusion and friction. These features are associated with deeper, more extensive lesions. Topical absorption may be limited and adherence reduced, while systemic treatments require careful balancing of efficacy and safety. Routine skincare may reduce adverse effects, improve adherence, and enhance topical outcomes.

**Conclusion:**

Management should consider skin‐specific differences. Clinicians should assess truncal involvement and select formulations that optimize permeability and usability. Future research should clarify truncal‐specific mechanisms and guide development of combination therapies and optimized topical formulations.

## 1 Introduction

Acne vulgaris is a common chronic inflammatory disorder of the pilosebaceous unit, affecting up to 80%–90% of adolescents and a considerable proportion of adults [1]. While most research and clinical guidelines focus on facial acne, truncal acne—lesions occurring on the chest, back, and shoulders—also represents a significant yet underrecognized disease burden [2]. Epidemiological studies indicate that approximately 50% of patients with facial acne have concurrent truncal involvement, and in some cases, truncal acne may occur independently [3, 4].

Despite its high prevalence, truncal acne has received limited attention in both research and clinical practice. This discrepancy may be attributed to the anatomical location of lesions, which are often concealed and underreported, as well as the difficulty in self‐assessment [5, 6, 7]. Although truncal and facial acne are generally believed to share similar pathogenic mechanisms, differences in skin physiology—including stratum corneum thickness, pilosebaceous unit density, sebaceous gland activity, skin microbiome composition, and exposure to external environmental factors—may influence lesion morphology, disease course, and treatment responsiveness [8, 9]. These variations have important implications for therapeutic decision‐making, particularly with regard to topical drug delivery, formulation design, and patient adherence.

In recent years, there has been growing recognition of the need to develop therapeutic strategies for truncal acne. However, comprehensive literature directly comparing truncal and facial acne in terms of physiological structure, clinical presentation, and treatment approaches remains scarce. This review aims to address this gap by: (1) summarizing the physiological differences between truncal and facial skin and their implications for acne development; (2) describing the clinical distinctions between truncal and facial acne; and (3) presenting comprehensive management strategies for truncal acne, including the efficacy and limitations of topical and systemic therapies, the significance of routine skin care, and a specific discussion of how formulation types influence daily acne management.

## 2 Methods

This study conducted a literature search of the PubMed and Google Scholar databases, including relevant English‐language publications up to December 2025. The search strategy combined anatomical site terms related to truncal and facial acne (e.g., “truncal acne,” “facial acne,” “back acne”), pathophysiology and microbiome‐related terms (e.g., “pathophysiology,” “sebum,” “microbiome,” “acne AND pH,” “body skin AND sweat glands”), as well as terms related to clinical manifestations, treatment, skin care, and formulations (e.g., “clinical manifestations,” “treatment,” “therapy,” “topical formulation,” “skincare,” “gel,” “cream,” “lotion,” “spray”), using Boolean operators (AND/OR) for combination. Literature selection was based on relevance to truncal acne and study quality to ensure scientific rigor and comprehensiveness.

### 2.1 Inclusion and Exclusion Criteria

Included studies comprised clinical research (randomized controlled trials, observational studies), cross‐sectional studies, and relevant reviews, focusing on facial or truncal acne pathophysiology, skin characteristics, microbiome composition, clinical manifestations, or topical/systemic treatment strategies, and also included studies on skincare and formulations. Excluded were non‐English publications, case reports, animal studies, and studies with unclear relevance to acne. Given the relative scarcity of literature on truncal acne, inclusion criteria regarding study type and methodological rigor were moderately relaxed to ensure comprehensiveness and representativeness of the review.

### 2.2 Literature Screening

Screening involved initial title and abstract review followed by full‐text assessment, conducted independently by the authors, with disagreements resolved through discussion. As this is a narrative review aiming to integrate current mechanistic and clinical evidence, no formal risk‐of‐bias assessment or quantitative analysis was performed.

## 3 Physiological and Structural Differences Between Truncal and Facial Skin and Their Functional Implications

At the physiological level, truncal and facial skin differ markedly in terms of thickness, stratum corneum structure, sebum secretion, microbiome composition, skin surface pH, and sweat gland distribution. These differences not only influence barrier function and the local microenvironment but may also modulate acne susceptibility, inflammatory patterns, and therapeutic responsiveness. Table 1 provides a systematic comparison of the major structural features of truncal versus facial skin. The following sections discuss the physiological significance of these differences in detail.

**TABLE 1:** Physiological and structural differences between facial and truncal skin.

Characteristic	Facial skin	Truncal skin	References
Stratum corneum thickness	9 ± 2 cell layers (thin)	13 ± 4 cell layers (slightly thicker^a^)	[10, 11]
Epidermal cell turnover rate	Fast	Slow	[10, 11]
Sebaceous gland density	400–900 glands/cm^2^	Usually less than 100 glands/cm^2^	[12]
Microbiota	Facial acne: higher *C. acnes* diversity	Truncal acne: Lower *C. acnes* diversity with a single dominant IA1 subtype; potentially influenced by cutaneous mycobiota components^a^	[13]
pH	5.4–5.6 (High)	5.0–5.2 (Lower)	[14]
Eccrine sweat glands	270 ± 25 glands/cm^2^	160 ± 15 glands/cm^2^	[15]

*Note:* Values represent approximate ranges reported in referenced anatomical and physiological studies.

^a^ Clinically actionable differences relevant for treatment planning.

### 3.1 Stratum Corneum Differences and Their Physiological Significance

The stratum corneum, the outermost layer of the epidermis, is composed of corneocytes at various stages of differentiation embedded in a hydrophobic lipid matrix, forming a “brick‐and‐mortar” structure [16]. This barrier protects against ultraviolet radiation, microbial invasion, and chemical insults, while reducing transepidermal water loss (TEWL) and maintaining skin homeostasis [17, 18].

Significant differences exist between truncal and facial skin with respect to stratum corneum thickness, corneocyte size, turnover rate, and TEWL. The facial stratum corneum is generally thinner, comprising approximately 9 ± 2 cell layers, though this varies with age, anatomical site, and individual factors [10]. Facial corneocytes are smaller and exhibit faster turnover, with a renewal cycle of around 1 week, albeit with some variation due to methodological differences and participant characteristics [19]. Correspondingly, TEWL is markedly higher on facial skin than on truncal skin, typically by 2–3‐fold. This higher permeability facilitates the absorption and bioavailability of topical agents—such as anti‐acne formulations—but also increases susceptibility to microbial colonization and environmental irritation.

In contrast, the truncal stratum corneum is generally thicker (average 13 ± 4 cell layers), with larger corneocytes and a relatively slower metabolic rate, exhibiting a renewal cycle of approximately 14 days [11]. While this provides a stronger barrier function, the reduced permeability may limit the transdermal delivery of topical therapeutics and, when follicular outflow is obstructed, may increase the risk of cyst and nodule formation [4, 20].

### 3.2 Sebum Secretion Differences and Their Physiological Significance

Sebum plays multiple roles in skin homeostasis, including lubricating and protecting the skin and hair, reducing transepidermal water loss, defending against microbial invasion, and maintaining skin softness and elasticity [21]. It is secreted by sebaceous glands, which are located between the hair follicle and arrector pili muscle and comprise a secretory portion and a duct. With the exception of certain regions such as the palms, soles, lips, and dorsal feet, sebaceous glands are distributed across almost the entire body surface [22].

Anatomically, sebaceous gland density on the face—including the forehead, cheeks, and chin—and the scalp typically ranges from 400 to 900 glands/cm^2^, whereas most other body sites generally exhibit densities below 100 glands/cm^2^; this density is influenced by factors such as age and sex [12]. Differences in gland density may directly affect both the quantity and composition of local sebum. In a study of 35 Korean female patients with acne, facial sebum secretion was generally higher than at truncal sites, with the highest levels observed in the T‐zone (up to 143.62 ± 57.19 μg/cm^2^). Within truncal regions, relatively higher sebum secretion was found at the midline chest (22.66 ± 18.16 μg/cm^2^) and the superior median upper back (23.57 ± 20.99 μg/cm^2^) [23]. Other studies have shown that increased facial sebum secretion is positively correlated with acne lesion counts [24, 25]. Excess sebum, together with follicular hyperkeratinization, can form comedones that obstruct hair follicles, creating a lipid‐rich anaerobic environment conducive to *Cutibacterium acnes* proliferation and secondary inflammation [26]. External stimuli such as ultraviolet radiation can further enhance sebum production and inflammatory responses [27]. Consequently, sebum secretion is considered a central pathogenic factor in facial acne [28].

In contrast, the trunk exhibits lower sebaceous gland density and secretion levels. Some studies suggest that the association between sebum production and truncal acne is less pronounced than that observed for facial acne, and that its pathogenesis may be more strongly influenced by mechanical factors [23]. The term “acne mechanica,” first proposed by Mills and Kligman in 1975, describes inflammatory acne induced by mechanical stimuli such as friction, pressure, traction, tight clothing, or occupation‐related rubbing [29]. Prolonged occlusion by clothing, creating a warm and humid microenvironment, may further exacerbate local inflammation [30]. Nevertheless, the potential contribution of sebum and its components to truncal acne cannot be excluded, particularly under conditions of elevated temperature, excessive sweating, or systemic hormonal influences.

In terms of composition, high‐density regions such as the face contain higher proportions of sebum‐specific lipids (e.g., squalene, C16:1 fatty acids) and certain epidermis‐specific lipids such as cholesterol sulfate (CHS), suggesting that sebaceous gland activity may influence epidermal lipid composition via the “cholesterol–CHS cycle” [31]. In low‐density regions such as the trunk, there is a greater abundance of stratum corneum‐derived lipids, including cholesterol and saturated long‐chain fatty acids (e.g., C24:0), whose levels are not significantly correlated with sebaceous gland density [31].

These compositional differences may modulate the local skin microenvironment and susceptibility to acne. For example, C16:1 fatty acid selectively inhibits *Staphylococcus aureus* activity [32]. Excessive squalene can enhance TREM2 macrophage phagocytosis of lipids and *C. acnes*, but its oxidized derivatives can induce keratinocytes to release pro‐inflammatory cytokines [33, 34]. CHS promotes profilaggrin production, thereby strengthening the skin barrier and maintaining water balance [35].

In summary, the distribution, secretion level, and composition of sebaceous glands vary considerably across body sites, shaping the local cutaneous microenvironment and influencing acne susceptibility. Facial skin is characterized by high gland density, active secretion, and enrichment of specific lipids, suggesting that acne management in this area may benefit from strategies aimed at reducing sebum production and limiting the pro‐inflammatory effects of sebum oxidation. In contrast, truncal acne involves a more complex pathophysiology, with existing evidence indicating that mechanical and environmental factors—such as sweat, sebum, pressure, friction, and skin occlusion—may contribute to its development and exacerbation, while microbial factors should also be considered.

### 3.3 Microbiome Differences and Their Physiological Significance

The composition of the skin microbiome differs substantially between truncal and facial acne. One study reported that, compared with healthy controls, facial acne samples exhibited increased relative abundances of *Staphylococcaceae*, *Enterococcus*, and unclassified *Planococcaceae*, whereas the relative abundance of *Propionibacterium* was decreased; in contrast, no significant differences in the relative abundances of *Propionibacteriaceae* or *Staphylococcaceae* were observed between acne and healthy groups in back samples [36]. Another study suggested that *Enterococcaceae* may represent a key bacterial contributor to truncal acne, potentially influenced by specific components of the cutaneous mycobiota [37].

Further studies indicate that the diversity of *Cutibacterium acnes* (*C. acnes*) strains on the trunk is lower than that on the face, with the IA1 subtype predominating in the back. This “microbial simplification” correlates with the severity of truncal acne [13]. Evidence suggests that the IA1 subtype exhibits higher immune activation, enhanced biofilm formation, and increased expression of virulence factors, which may drive deeper inflammatory responses in truncal lesions [38]. Conversely, facial acne typically harbors multiple *C*. *acnes* genotypes (e.g., IA1, IA2, IC, II), and inflammation is often more superficial [39]. These differences provide a rationale for exploring site‐specific, personalized treatment strategies.

### 3.4 Differences in pH and Sweat Gland Distribution and Their Physiological Significance

Truncal and facial skin also differ in pH and sweat gland distribution, which may significantly influence acne development and the local microenvironment. Acidic conditions promote keratinocyte maturation and differentiation, enhance epidermal lipid formation, and inhibit pathogenic microorganisms, thereby maintaining skin health [40, 41]. Facial skin typically has a pH of 5.4–5.6, slightly higher than truncal skin (5.0–5.2), with a stable linear correlation between the two, suggesting that regional skin pH may be systemically regulated [14]. Facial pH negatively correlates with sebum secretion, whereas truncal pH shows no clear relationship with sebum output, which may imply that truncal pH is influenced by non‐sebaceous factors [14, 42]. Elevated skin pH has been reported to increase acne risk and recurrence [40]. Overall, facial acne management may rely more on sebum regulation, while truncal acne may require consideration of pH‐modulating factors [43].

Regarding sweat glands, facial skin exhibits a higher density (approximately 270 ± 25 glands/cm^2^) compared with truncal skin (approximately 160 ± 15 glands/cm^2^), although some variation may occur between individuals and anatomical sites [15]. Despite the lower sweat gland density per unit area, the trunk has a large surface area, and sweat accumulation under clothing may create a warm and humid microenvironment that may promote mechanical acne and fungal colonization [44, 45]. However, a single‐blind randomized pilot study reported no significant correlation between exercise‐induced sweat or desquamation and truncal acne severity, suggesting that these effects may be influenced by study population characteristics, environmental conditions, and assessment methods [46].

## 4 Clinical Presentation Differences

Facial acne presents with a spectrum of lesions, including open comedones (blackheads), closed comedones (whiteheads), inflammatory papules, pustules, nodules, and cysts, corresponding to different pathological stages and severities [43].

In contrast, truncal acne is predominantly inflammatory, with common lesions including papules, pustules, nodules, and cysts. In severe cases, multiple lesion types may coexist, indicating deeper inflammation and greater disease severity (see Table 2) [47, 48]. Truncal acne is more likely to result in scarring, particularly on the shoulders and upper chest, and in some cases can lead to permanent, severe atrophic or hypertrophic scars [51]. Approximately 10% of patients with truncal acne develop scarring, which may be associated with higher collagen content and fibroblast fibrotic activity in truncal skin [53, 54]. Post‐inflammatory hyperpigmentation (PIH) is also more frequent and persistent on the trunk, likely due to deeper inflammation, larger affected areas, and thicker skin [48].

**TABLE 2:** Clinical differences between facial and truncal acne.

Characteristic	Facial acne	Truncal acne	References
Inflammation severity	Relatively mild	More severe, prone to pustules and cysts	[47, 48]
Surface area affected	Small	Extensive^a^	[48]
Post‐inflammatory hyperpigmentation	Depends on severity	Higher risk, longer duration^a^	[48]
Acne type	Vulgar acne is more common	Acne conglobata and acne fulminans may be observed	[43, 49, 50]
Acne scarring	Less common, predominantly atrophic scars	Hypertrophic scars (e.g., keloids) more frequent^a^	[51]
Other considerations	—	Differential diagnosis should include Malassezia folliculitis^a^	[52]

^a^ Clinically actionable differences important for treatment planning.

Truncal acne may also present as two rare but severe variants. Acne conglobata (AC) is characterized by interconnected deep abscesses, most frequently involving the shoulders, chest, upper arms, and buttocks. The cysts often contain malodorous purulent material and may lead to disfiguring scarring. Some cases have been linked to the use of androgenic anabolic steroids [49, 55]. Acne fulminans (AF) manifests acutely with painful, ulcerative, and hemorrhagic lesions, predominantly in males. These lesions frequently exhibit resistance to conventional antibiotic therapy and may be precipitated by high‐dose isotretinoin; in some patients, elevated serum testosterone levels have been observed [50, 56].

It is important to note that inflammatory papules or pustules on the trunk in young patients are not always acne; they may represent *Malassezia* folliculitis, which lacks comedones or other obstructive lesions and typically presents as dimorphic eruptions, including pruritic papules and pustular follicular lesions. Differentiation can be achieved through mycological examination (e.g., KOH smear) or skin biopsy, both of which may reveal numerous round budding yeast cells [52]. Accurate diagnosis is essential for appropriate treatment.

## 5 Comprehensive Management Strategies for Truncal Acne

Truncal acne differs from facial acne in both pathophysiology and clinical features. Its main characteristics include a thicker stratum corneum, lower sebaceous gland density, a relatively less diverse microbiota, and widespread lesions predominantly of inflammatory type. In addition, mechanical factors such as clothing friction, sweat retention, and pressure may exacerbate the condition. These factors contribute to a potentially longer treatment course. Therefore, alongside topical or systemic therapies, reinforcing daily skincare and modifying relevant lifestyle factors are particularly important.

### 5.1 Topical Therapy

#### 5.1.1 Retinoids

Topical retinoids (tretinoin, adapalene, tazarotene, trifarotene) remain the cornerstone for treating both non‐inflammatory and inflammatory acne by regulating keratinization, preventing microcomedo formation, and suppressing inflammation.

Trifarotene is the first topical retinoid with high selectivity for retinoic acid receptor γ (RAR‐γ) that has been rigorously demonstrated to be safe and effective for truncal acne in clinical studies [57]. Topical 0.045% tazarotene has also shown clinical improvement in moderate truncal acne [58]. Common adverse effects include erythema, pruritus, dryness, and irritation, most of which can be alleviated through gradual tolerance induction or adjunctive moisturization [59].

#### 5.1.2 Benzoyl Peroxide (BPO)

BPO plays an important role in acne management due to its antimicrobial activity, which effectively reduces antibiotic‐resistant *C. acnes* [60]. Studies have shown that both leave‐on and short‐contact BPO formulations can decrease *C. acnes* colony counts on the back [61, 62, 63]. The main adverse effect of BPO is its bleaching property, which may cause discoloration of clothing, bedding, or hair, particularly when applied over large areas. To maintain efficacy while minimizing this effect, foam or rinse‐off formulations are generally preferred for truncal application [64].

#### 5.1.3 Topical Antibiotics

Topical antibiotics (e.g., clindamycin, erythromycin, minocycline) inhibit *C. acnes* growth and possess anti‐inflammatory properties. However, monotherapy carries a risk of antibiotic resistance, and these agents are therefore commonly combined with BPO or topical retinoids to enhance efficacy and reduce resistance [65]. While topical antibiotics are widely used for facial acne, their effectiveness in truncal acne remains inadequately documented. A recent pooled post hoc analysis indicated that a fixed‐dose triple‐combination gel containing clindamycin, adapalene, and BPO (CAB) demonstrated good tolerability on the trunk over a 12‐week treatment period, with mostly mild adverse events, suggesting potential applicability in this region [66].

#### 5.1.4 Azelaic Acid

Azelaic acid is available both as a topical medication and in cosmetic formulations. It exerts multiple effects, including normalization of abnormal keratinization, anti‐inflammatory and antimicrobial activity, and reduction of post‐inflammatory hyperpigmentation, making it suitable for both inflammatory and non‐inflammatory acne. Commonly used concentrations are 15% or 20%, with generally mild adverse effects such as erythema, pruritus, scaling, and burning sensation [67]. Given its low percutaneous absorption, novel delivery systems such as liposomes and microemulsions have been developed to improve penetration and tolerability [68]. A small study (*n* = 18) reported that 15% azelaic acid foam applied twice daily on moderate truncal acne resulted in significant improvement with good tolerability [69].

#### 5.1.5 Limitations of Topical Therapy

Topical treatments for truncal acne have several limitations:

1. A thicker stratum corneum reduces percutaneous penetration;
2. Large lesion areas complicate application;
3. Local irritation and bleaching effects may reduce adherence;
4. Insufficient control of moderate‐to‐severe inflammatory lesions.

These factors may lead some patients to prefer systemic therapy over topical agents [70]. Moreover, for moderate‐to‐severe inflammatory lesions, topical therapy alone is often inadequate, necessitating combination with systemic treatments and standardized skincare measures to achieve optimal outcomes.

### 5.2 Systemic Therapy

Systemic therapy is indicated for moderate‐to‐severe inflammatory acne or cases with limited response to topical treatment and is usually combined with baseline topical regimens to enhance efficacy and reduce the risk of resistance.

#### 5.2.1 Systemic Retinoids

Isotretinoin exerts its effects by inhibiting sebum production, regulating follicular keratinization, and inducing sebaceous gland apoptosis, making it the first‐line systemic agent for severe or refractory truncal acne [71]. Adverse effects are dose‐dependent, with common adverse effects including skin and mucosal dryness and cheilitis (incidence up to 90%–100%), while serious adverse effects include teratogenicity. The association between isotretinoin and depressive symptoms remains controversial [72]. Standardized dosing regimens should be followed, with regular monitoring of laboratory parameters and psychological status to ensure both efficacy and safety [73].

#### 5.2.2 Systemic Antibiotics

Systemic antibiotics are widely used for moderate‐to‐severe acne, with tetracyclines (e.g., doxycycline, minocycline) being the most commonly prescribed due to their combined antibacterial and anti‐inflammatory effects [74]. Broad‐spectrum antibiotics, however, may induce off‐target effects and gastrointestinal adverse reactions [75]. Recently, the narrow‐spectrum antibiotic sarecycline has shown promising efficacy in truncal acne. Multiple phase III randomized controlled trials demonstrated that sarecycline significantly improved moderate‐to‐severe truncal acne after 12 weeks of treatment with a favorable safety profile [76, 77, 78]. Open‐label studies further suggest that sarecycline can achieve marked, and in some cases near‐clearance, clinical improvement while simultaneously alleviating symptoms and psychological burden in a proportion of patients [79].

#### 5.2.3 Systemic Hormonal Therapy

In adult female patients, systemic hormonal therapy represents an effective alternative to antibiotics, particularly in cases where acne fluctuates with the menstrual cycle or is associated with endocrine disorders such as polycystic ovary syndrome [80]. Combined oral contraceptives (COCs) improve acne by suppressing ovarian androgen production, though clinical effects typically require 3–6 months to manifest [81]. Small‐scale randomized double‐blind studies have shown that drospirenone/ethinyl estradiol‐containing formulations significantly reduce both inflammatory and non‐inflammatory truncal lesions over 24 weeks with good tolerability, although lesion clearance rates did not reach statistical significance compared with placebo. Common adverse effects include breast tenderness, mood changes, gastrointestinal discomfort, weight gain, headache, and breakthrough bleeding [82].

Spironolactone, an anti‐androgenic diuretic, inhibits sebum production by blocking androgen receptors and is a commonly used systemic therapy in adult women [83]. A retrospective study including 403 patients reported improvement rates of 84.0% for chest acne and 80.2% for back acne. Adverse effects included menstrual irregularities, diuresis, fatigue, and headache, but overall tolerability was favorable [84]. However, as this study was retrospective, its efficacy specifically for truncal acne requires confirmation in prospective trials.

#### 5.2.4 Limitations of Systemic Therapy

The main limitations of systemic therapy include antibiotic resistance, isotretinoin‐associated teratogenicity and mucosal dryness, and the limited applicability of hormonal treatments. Strict monitoring is required to balance efficacy and safety.

### 5.3 Daily Skincare for Truncal Acne

Adherence has been reported as an important factor contributing to treatment failure in truncal acne [85]. Appropriate daily skincare can reduce drug‐related adverse effects, improve patient compliance, and may also optimize the penetration and efficacy of topical agents [86].

#### 5.3.1 Cleansing

Cleansing is a fundamental step in daily skincare, aiding in the removal of sweat, sebum, and accumulated corneocytes, thereby reducing follicular occlusion and the risk of microcomedone formation [87]. A single‐center, open‐label, non‐randomized study (*n* = 51) evaluated the daily use of a deep‐cleansing gel containing 2% salicylic acid, 0.2% zinc gluconate, and 0.05% lipohydroxy acids over 84 consecutive days in patients with mild‐to‐moderate truncal acne. The treatment resulted in a 56.3% reduction in total lesion count, with inflammatory lesions decreasing by 48.2% and non‐inflammatory lesions by 64.0%. In addition, skin barrier function improved, as indicated by a 21.3% decrease in TEWL [88]. Another four‐week clinical study (*n* = 29) similarly demonstrated that cleansing and exfoliating products could improve truncal acne lesions, although these findings require validation in larger randomized controlled trials [89]. As these studies were open‐label and non‐randomized, their findings require validation in larger randomized controlled trials.

Although cleansing products have demonstrated efficacy, appropriate use is crucial. Over‐cleansing or the use of harsh soap‐based surfactants should be avoided to prevent barrier disruption and exacerbation of inflammation [90, 91]. High‐temperature water and vigorous rubbing of affected areas should also be avoided to minimize mechanical irritation. Multiple studies have shown that mildly acidic cleansers are advantageous for improving acne lesions and maintaining skin tolerance [92].

#### 5.3.2 Anti‐Acne Body Care Products

Anti‐acne body care products are designed to act through four main mechanisms: promotion of keratin exfoliation, regulation of sebum production, antimicrobial activity, and reduction of inflammation.

##### 5.3.2.1 Promotion of Keratin Exfoliation

Exfoliation helps remove aged corneocytes and correct abnormal follicular keratinization, contributing to the improvement of truncal acne and enhancing the penetration and absorption of subsequent active ingredients [93]. Depending on their mechanism of action, exfoliating agents are generally classified into physical, chemical, or enzymatic types. Table 3 compares the different types of exfoliation and their respective applications.

**TABLE 3:** Comparison of exfoliation methods and recommendations.

Type	Representative agents	Mechanism of action	Advantages	Limitations	Recommendations for use in truncal acne	References
Physical exfoliation	Abrasive particles, etc.	Promotes corneocyte removal through mechanical friction	Direct action, rapid onset	May cause micro‐injuries and trigger or exacerbate inflammation	Not recommended for routine use; suitable only for non‐inflamed skin	[93]
Chemical exfoliation	α‐Hydroxy acids (glycolic acid, lactic acid, mandelic acid); salicylic acid	Weakens intercellular adhesion and promotes desquamation	Facilitates corneocyte turnover; generally milder than physical exfoliation	May cause irritation; tolerance needs to be established	Can be used at low concentrations if tolerated; monitor irritation and adjust frequency accordingly	[94]
Enzymatic exfoliation	Papain, bromelain	Degrades keratin proteins and desmosomal structures to promote exfoliation	Relatively low irritation	Enzymes are prone to inactivation; formulation stability must be ensured	More suitable for sensitive skin or patients with lower tolerance in truncal acne	[95]

Physical exfoliants (e.g., abrasive particles) promote corneocyte removal through mechanical friction but may cause micro‐injuries to the epidermis and induce inflammation. Given that truncal acne lesions often exhibit varying degrees of inflammation, physical exfoliation is generally not recommended as a routine care method and should only be used cautiously on non‐inflamed skin [93].

Chemical exfoliants primarily include α‐hydroxy acids (AHAs, such as glycolic, lactic, and mandelic acids) and salicylic acid. AHAs promote exfoliation by reducing epidermal calcium concentration and weakening corneocyte adhesion. Low pH and higher concentrations of AHAs generally produce more pronounced exfoliation but may also increase skin irritation [94]. In cosmetic formulations, AHA concentrations typically do not exceed 10%, and in certain countries, such as China, the maximum permitted concentration is 6% [96]. Therefore, formulation design must balance acid concentration and pH to optimize individual tolerability and desired efficacy. Salicylic acid, a lipophilic β‐hydroxy acid, can dissolve intercellular lipids, promote corneocyte turnover, and soften comedones, making it particularly suitable for oily and acne‐prone skin [97, 98]. Similar to AHAs, individual tolerance should be considered when using salicylic acid.

Enzymatic exfoliants (e.g., papain and bromelain) promote keratin removal by degrading keratin proteins and desmosomal structures, generally exhibiting lower irritation and thus considered suitable for individuals with more sensitive skin [95]. However, enzymatic agents are susceptible to inactivation by external factors, and formulation stability must be carefully addressed in practical applications.

##### 5.3.2.2 Sebum Regulation

Although sebum production may not be the primary pathogenic factor in truncal acne, its role should not be overlooked [23]. Several sebum‐controlling agents have been identified: epigallocatechin gallate (EGCG) can induce apoptosis of SEB‐1 sebocytes, thereby reducing sebum secretion [99]; zinc salts inhibit 5α‐reductase activity and lower dihydrotestosterone levels, contributing to sebum control [100]; Isosilybin modulates lipid synthesis and oxidation via the AMPK/SREBP‐1c/PPARα signaling pathway [101]; and tetrahydrocurcumin promotes terminal sebum degradation by upregulating lipophagy‐related signals (e.g., LC3, p62) and enhancing the expression of lipolytic enzymes (e.g., ATGL, HSL, MGL) [102]. Natural mineral‐based ingredients, such as bentonite, diatomaceous earth, and montmorillonite, possess high specific surface areas and strong adsorption capacities, making them widely applied in oil‐control skincare formulations [103].

##### 5.3.2.3 Antimicrobial Activity

In the management of truncal acne, attention should be given not only to acne‐causing bacteria such as *Cutibacterium acnes* but also to Malassezia, an important pathogen implicated in folliculitis. Due to their overlapping clinical manifestations, these microorganisms are difficult for the general consumer to distinguish. Therefore, anti‐acne cosmetic formulations should be designed to inhibit both *C. acnes* and *Malassezia* [104, 105]. Common agents that suppress *C. acnes* include quaternary ammonium compounds (e.g., Quaternium‐73) and *Myrtus communis* fruit extracts, while Tea Tree Oil exhibits dual activity against both *C. acnes* and *Malassezia* [106]. These ingredients are widely incorporated into anti‐acne skincare products, providing an effective basis for truncal acne management.

##### 5.3.2.4 Anti‐Inflammatory and Soothing Effects

Truncal acne is often accompanied by inflammation; compared with facial acne, greater emphasis should be placed on anti‐inflammatory and soothing interventions. Niacinamide (vitamin B3 amide) is a well‐established anti‐inflammatory agent that can reduce sebum secretion, improve skin barrier function, and exert skin‐lightening effects [107]. A double‐blind randomized clinical trial demonstrated that 5% niacinamide gel was comparable in efficacy to 2% clindamycin gel for the treatment of mild‐to‐moderate acne, with good tolerability and no significant adverse effects; its anti‐inflammatory activity can improve inflammatory lesions and reduce post‐acne erythema and inflammatory hyperpigmentation [108]. Other agents, including quercetin, lavender essential oil, and plant extracts such as *Centella asiatica* and dipotassium glycyrrhizate, have also been shown to reduce inflammation and improve acne lesions [109]. One study indicated that a topical plant‐based skincare regimen containing *Centella asiatica* and related ingredients could simultaneously reduce mild‐to‐moderate facial and truncal acne lesion counts while enhancing patients' positive mood [110]. Furthermore, novel biotechnological plant complexes, such as *Camellia sinensis* callus lysate, have demonstrated reductions in erythema and scaling, thereby reducing lesion severity in mild‐to‐moderate truncal acne [43].

#### 5.3.3 Photoprotection

Ultraviolet (UV) exposure can exacerbate inflammation in acne, prolong lesion healing, and promote post‐inflammatory hyperpigmentation (PIH) [111]. Moreover, commonly used acne treatments, such as vitamin A derivatives and benzoyl peroxide, may increase photosensitivity. Appropriate photoprotection can mitigate UV‐induced skin damage, reduce treatment‐related adverse effects, and enhance overall therapeutic tolerability. Therefore, for patients with truncal acne, it is recommended to apply adequate sun protection to exposed skin areas—particularly in Asian populations—to lower the risk of UV‐induced damage and PIH [112]. When selecting sunscreens, formulations that are lightweight and non‐comedogenic should be prioritized.

#### 5.3.4 Formulation Characteristics for Daily Care of Truncal Acne

Topical formulations do not alter the pharmacological activity of active ingredients but can significantly affect percutaneous penetration, local concentration, skin tolerability, and patient adherence. Common formulations used for daily care of truncal acne include creams, lotions, gels, foams, and sprays, with their respective characteristics and recommended application summarized in Table 4.

1. Creams: Oil‐in‐water or water‐in‐oil emulsions with strong moisturizing properties and a gentle, non‐irritating texture, suitable for dry or sensitive skin. For patients with oily skin, creams may feel greasy and reduce adherence [113].
2. Lotions: Water‐based emulsions with a lightweight texture that are easy to apply, particularly over large or hair‐covered areas. During water‐phase evaporation, lotions can provide a cooling effect, but their moisturizing and occlusive properties are lower than those of creams [114].
3. Gels: Semi‐solid formulations that form a non‐oily, non‐occlusive film upon drying, allowing easy application and removal, particularly suited for oily or inflamed areas. Gels provide a cooling sensation but limited hydration, are prone to being washed off by sweat, and may stick to clothing. Prolonged coverage on the trunk may affect drug stability [115]. Compared with creams, gels generally offer higher percutaneous penetration; for example, 15% azelaic acid gel exhibits approximately eightfold greater skin penetration than 20% azelaic acid cream [69].
4. Foams: Composed of active ingredients, propellants, and surfactants, foams disperse quickly over the skin with minimal mechanical friction, making them suitable for sensitive or inflamed regions. Although non‐occlusive and providing limited hydration, foams can rapidly cover large areas, enhancing local absorption and patient adherence. Some formulations may induce mild stinging or burning on damaged skin [116].
5. Sprays: Pressurized liquid formulations capable of covering large surface areas (approximately 15%–20% of body surface area), especially suitable for difficult‐to‐reach areas such as the trunk, thereby improving adherence. Sprays leave minimal residue, reduce cross‐contamination risk, and provide a cooling sensation. Caution is required in skin folds due to higher absorption, and mild stinging or burning may occur in some cases [117, 118].

**TABLE 4:** Characteristics and recommended applications of topical formulations for truncal acne.

Formulation	Key features	Limitations	Recommended use	References
Cream	Oil‐in‐water or water‐in‐oil, thick texture, strong moisturizing	May feel greasy on oily skin, slower absorption	Dry or sensitive skin	[113]
Lotion	Water‐based, lightweight, easy to apply	Lower moisturizing and occlusive properties than creams	Large or hair‐covered areas	[114]
Gel	Semi‐solid, forms non‐oily, non‐occlusive film, refreshing texture	Limited hydration, easily washed off by sweat, may stick to clothing	Oily or inflamed skin, hair‐covered areas	[115]
Foam	Pressurized liquid with active ingredients, propellants, surfactants, spreads easily with minimal friction	Non‐occlusive, limited hydration, may sting or burn on damaged skin	Sensitive or inflamed skin, large or hair‐covered areas	[116]
Spray	Pressurized liquid, lightweight, fast absorption, broad coverage	High absorption in skin folds, may sting or burn	Trunk and large affected areas; caution in skin folds	[117, 118]

### 5.4 Impact of Lifestyle and Dietary Habits

Current evidence indicates that various lifestyle and dietary factors are associated with the onset and severity of acne vulgaris, including dietary patterns, endocrine status, psychological stress, smoking, and physical activity [119]. In individuals with truncal acne, a self‐reported survey conducted in France involving 1001 adolescents and young adults found that psychological stress (46.3%), high‐fat diet (33.2%), and insomnia (27.0%) were perceived by participants as the main triggering factors for truncal acne. Although this study had methodological limitations, including an imbalanced sex ratio, lack of objective dermatological diagnosis, and subjective assessment of acne severity, the results provide valuable supplementary information regarding potential patient‐perceived contributing factors for truncal acne [7].

Moreover, studies suggest that wearing loose, breathable cotton clothing and promptly cleansing sweat and changing garments after exercise may help improve clinical outcomes in truncal acne patients [42]. However, a small pilot study indicated that exercising five times per week with showers at different times had no significant effect on the incidence of truncal acne (*p* > 0.05), suggesting that sweat elimination and exercise by themselves may have limited impact on truncal acne. Given the small sample size of this study (7–8 participants per group), the short intervention period, and the lack of long‐term follow‐up, these findings should be interpreted with caution [46].

## 6 Conclusions

Facial and truncal acne differ in certain aspects of pathophysiology and clinical presentation, which have important implications for diagnosis and management. Truncal acne is typically associated with a thicker stratum corneum, lower sebaceous gland density and activity, broader lesion distribution, and frequent mechanical influences such as friction and occlusion from clothing. These features may contribute to deeper inflammatory involvement, a higher risk of scarring, and reduced responsiveness to conventional topical therapies due to limited transdermal penetration.

From a clinical perspective, truncal involvement should be actively assessed in patients presenting with facial acne, as it is often under‐recognized. In addition, the selection and usability of topical vehicles play an important role in truncal acne management, particularly in the setting of suboptimal patient adherence. Topical formulations that facilitate application over large or difficult‐to‐reach areas may help improve treatment coverage and adherence.

Future research should prioritize elucidating the molecular mechanisms and microbiome characteristics specific to truncal acne and systematically evaluate combined therapeutic strategies targeting sebum regulation, microecological modulation, and inflammatory control through well‐designed, large‐scale clinical trials. Furthermore, the development of optimized topical formulations with improved penetration and tolerability, together with evidence‐based lifestyle and dietary interventions, represents a promising direction for advancing the comprehensive management of truncal acne.

## Author Contributions

**Xiaoyue Feng:** writing – review and editing, writing – original draft, visualization, methodology, investigation. **Yong Chen:** validation, methodology, investigation. **Youting Liu:** supervision, methodology, conceptualization.

## Funding

This study was not supported by any sponsor or funder.

## Ethics Statement

The authors have nothing to report.

## Consent

The authors have nothing to report.

## Conflicts of Interest

The authors declare no conflicts of interest.

## Data Availability

No unpublished data, proprietary industry materials, company documents, or internal technical information were used in the preparation of this narrative review. All information discussed in the manuscript is derived from publicly available, peer‐reviewed literature.
